# The Impact of Short-Form Video and Optimistic Bias on Engagement in Oral Health Prevention: Integrating a KAP Model

**DOI:** 10.3390/bs14100968

**Published:** 2024-10-18

**Authors:** Donghwa Chung, Jiaqi Wang, Yanfang Meng

**Affiliations:** 1 School of Journalism and Communication, Central China Normal University, Wuhan 430079, China; chungdonghwa@ccnu.edu.cn (D.C.); wangjiaqi1004@126.com (J.W.); 2 School of Journalism and Communication, Beijing Institute of Graphic Communication, Beijing 102699, China

**Keywords:** short-form video exposure, Douyin, KAP theory model, optimistic bias, oral health prevention, college students, survey

## Abstract

College students are recognized as a demographic particularly susceptible to higher oral health risks due to irregular and unhealthy habits. While previous research has underscored these risks through descriptive studies within this group, a significant gap persists in the literature regarding the impact of contemporary media, specifically oral-health-related content, on the engagement in oral health prevention among Chinese college students. To address this gap, this study, guided by the extended knowledge–attitude–practice (KAP) theory model, explores the direct, mediated, and serially mediated mechanisms through which exposure to oral health short-form videos on Douyin influences their participation in oral health prevention. An empirical cross-sectional online survey was conducted, with valid data (N = 421) analyzed using descriptive statistics and hierarchical regression. Mediation and serial mediation analyses were also performed using SPSS version 25.0. The analysis revealed that exposure to oral health short-form videos had a positive direct effect on both oral health knowledge and the reduction of optimistic bias among Chinese college students. Furthermore, a significant serial mediation effect of oral health knowledge and oral health attitude was identified. The findings underscore the substantial applicability and explanatory power of the extended KAP theory model in understanding engagement in oral health prevention.

## 1. Introduction

Oral diseases are a growing global public health problem, affecting more than 3.5 billion people worldwide [1]. These diseases pose significant risks to individual health, leading to conditions such as oral pain (e.g., tooth decay and gum inflammation), chronic diseases (e.g., diabetes and cardiovascular disease), and potentially fatal cancers [2,3,4]. Although most oral diseases can be prevented and treated early through self-care practices, such as toothbrushing and regular dental check-ups [5,6,7,8], their prevalence remains high. Furthermore, despite extensive oral health promotion efforts [9], the willingness to engage in preventive oral health practices remains relatively low, especially in low-income countries [10]. Considering this situation, healthcare providers and researchers have been calling for further investigation into the factors that influence individuals’ participation in preventive practices [11,12].

To date, there has been a significant reduction in the incidence of oral diseases, and a significant impact on Chinese citizens’ engagement in oral health prevention (EOHP), such as avoiding sweets and fizzy drinks, practicing oral hygiene, and visiting the dentist regularly [13,14]. One possible reason for this success is the significant contribution of oral health promotion on media platforms. In China, short-form video platforms have gained considerable popularity and have changed the way people access health-related information [15,16,17]. Douyin, specifically, has become the most widely used platform across all age groups in China [18]. Its unique features, including the ability to create, view, and share short-form video content, make it an essential tool for both entertainment and education, especially in oral health promotion [19,20,21]. In particular, oral health content uploaded by health institutions and dental professionals receives significant engagement in terms of likes and shares [22]. Previous studies have shown that frequent exposure to health-related short videos can encourage individuals to adopt healthier behaviors [23]. However, there remains a gap in the literature to explore the effects of exposure to oral health short-form videos (EOHSFV) via Douyin on engagement in oral health prevention (EOHP) in the Chinese context.

Although recent studies have examined the impact of oral-health-related social media content on individuals’ perspectives [24,25], several limitations remain. First, most research has focused on factors that influence behavior, such as cognitive and psychological aspects. However, there is a notable lack of application of theoretical frameworks, such as psychological bias theories, to comprehensively examine these complex effects. Second, while numerous scholars have used the knowledge, attitude, and practice (KAP) theory to examine various health concerns [26,27,28], there is a dearth of research that extends the applicability and explanatory power of the KAP model by incorporating key concepts, such as media exposure and other psychological factors. Finally, although recent studies have examined factors influencing oral health behaviors in adolescents [25], parents [29], and pregnant women [30], there is a lack of research focused on college students as a specific demographic. Additionally, college students are a critical demographic to target due to their tendency toward irregular eating habits, such as consuming foods high in sugar and carbohydrates, and their limited access to screening and early intervention [31]. Identifying these challenges is crucial for designing effective health strategies for this demographic. Therefore, exploring the impact of new media on their knowledge, attitudes, and behaviors is essential to address this research gap.

As mentioned above, the present study, guided by the KAP theory and optimistic bias theory, aims to investigate both the direct and indirect effects of EOHSFV on EOHP among Chinese college students aged 18 to 24 years.

## 2. Literature Review

### 2.1. Extended KAP Theory Model

The KAP model, first proposed by Hochbaum [32], suggests that individuals’ engagement in health practices is primarily determined by their attitudes and knowledge [33,34]. For example, a recent study showed that individuals’ vaccination behavior was influenced not only by their knowledge about vaccines, but also significantly by their attitudes toward vaccination [35]. As a leading health behavior framework, the KAP model has demonstrated its robustness and high applicability in various studies [34,36,37]. However, recent studies have criticized the KAP model for its limited explanatory power regarding the variance of health behaviors [38]. A previous study pointed out that the KAP model does not address broader predictors of health practices that could lead to the development of more comprehensive frameworks that better explain individuals’ health behaviors [39]. In recent years, efforts to improve the explanatory and predictive capabilities of the KAP model have attracted the attention of health promotion scholars [39,40,41]. Health scholars have extended the KAP model by adding factors such as media exposure, injunctive norms, and perceived threat to better understand individuals’ antibiotic use [39] and intention to receive the HPV vaccine [41]. These studies have practical implications for designing effective interventions to improve health outcomes.

In addition to these extended factors, psychological biases have received considerable attention in the study of health behaviors and practices. For example, previous studies have found that people tend to overestimate the likelihood of experiencing positive outcomes in the future and underestimate the likelihood of experiencing negative events [42]. Such a psychological bias, known as the “optimistic bias”, has been recognized as a key determinant of individuals’ health perceptions and behavioral decisions [43,44]. The health behavior literature suggests that individuals’ optimistic bias can both positively and negatively influence their engagement in preventive health behaviors, such as practicing hand hygiene [45] and refusing to quit vaping [46]. Among the relevant empirical studies, reversed optimistic bias (ROB) stands out as a critical antecedent of proactive health prevention. In this study, ROB is defined as the tendency for individuals to assume that they are more likely to experience harmful oral health outcomes than others.

In a pioneering public-health-related study, the determinants of foodborne illness were examined using the KAP model and the concept of optimistic bias. However, the results showed that optimistic bias did not significantly influence attitudes or practices in the Brazilian context [47]. There is a notable gap in the oral health field, as no study has yet used the KAP model and optimistic bias theory to explore oral health prevention. Therefore, the current study aims to develop a comprehensive framework by integrating the extended KAP theory model with optimistic bias. This framework will be used to examine the effect of EOHSFV on Chinese college students’ EOHP.

### 2.2. EOHSFV on KAP Constructs and ROB

Douyin (the Chinese version of TikTok) is recognized as a leading communication platform, with approximately 400 million daily active users in China [48]. Douyin provides engaging user experiences that satisfy social needs, such as making new friends and virtual interactions [49]. As one of the most widely used platforms among Chinese young adults [49], Douyin is also an essential source of health information, providing treatment options and medical advice for chronic disease management [50]. A recent report highlighted efforts by the National Health Commission (NHC) and other health authorities to improve health promotion through short-form video platforms. The report, China Health Communication 2024, showed that NHC health accounts are prominent in creating health content on popular Chinese social media platforms, with 80.6% of health accounts on Douyin. In addition, 45% of short-form health promotion videos were created by NHC accounts [31].

As Douyin continues to expand its health promotion efforts, covering various preventive measures, oral health content in particular has received significant attention from young adult users. In addition, a review of previous research found that a number of studies evaluated the reliability of oral-health-related content on Douyin [24,25]. However, there was a lack of attention to exploring how exposure to such content may improve oral health behaviors among Chinese young adults and to identifying key mediating mechanisms in this relationship. Investigating this effect is critical to understanding the implications for preventive behaviors among Chinese young adults. In the current study, EOHSFV is defined as the frequency with which Chinese young adults view oral health prevention-related content on Douyin, including practical tips for protecting teeth, educational videos on oral health knowledge, and animated videos on oral health education.

A considerable number of health promotion studies have applied the KAP model to examine individuals’ knowledge, attitudes, and practices regarding their participation in health prevention [33,34,35]. Knowledge was defined as the understanding of information, which is the conscious and non-symbolic perception of meaning [51]. Moreover, this factor only evaluates the individual’s knowledge of public health concepts related to national and international public health strategies [33]. In the current study, oral health knowledge (OHK) refers to an individual’s awareness and understanding of the circumstances and preventive practices necessary to maintain oral health, such as keeping teeth and gums healthy. Attitude stands out as a key factor of OHK, which has been found to have positive correlations with various health behaviors, as substantiated by multiple studies; for example, managing diet [52], smoking cessation [53], and COVID-19 prevention [54]. A previous study referred to health attitudes as an individual’s feelings and perspectives toward health prevention. Specifically, it addressed how individuals emotionally perceive health-related issues and recommended health behaviors. In addition, it assessed how people’s engagement in preventive health measures is influenced by their attitudes toward preventing health problems [55]. In the current study, oral health attitude (OHA) is defined as an individual’s positive or negative evaluation of engaging in oral health prevention.

Previous literature has demonstrated that exposure to health information is a strong antecedent of cognitive factors, such as knowledge. For instance, Cameron et al. found that individuals exposed to information about influenza significantly increased their knowledge about influenza and its vaccination [56]. Similarly, public health scholars found a positive association between exposure to COVID-19-related news and individuals’ knowledge of preventive measures [57]. Additionally, a prior study found that exposure to breast-cancer-specific information increased individuals’ knowledge of the harmful effects of smoking [58].

Earlier studies also investigated the impact of media on psychological biases. Specifically, a considerable number of studies found that individuals’ reversed optimistic bias (ROB) was influenced by exposure to different types of information, such as HIV prevention videos and cancer prevention advertisements [59,60]. For example, previous research found that when individuals are exposed to health messages regarding cancer risk and prevention, they are more likely to perceive these messages as having a greater impact on themselves than on others [60]. In other words, the more frequently individuals are exposed to health information, the more likely they tend to assume that they are more likely to experience harmful health outcomes than others (ROB). Similarly, a previous study found that exposure to climate-change-related messages increased individuals’ levels of the first-person effect [61]. This suggests that exposure to information is negatively associated with perceptions of invulnerability compared to others (ROB). Therefore, the following hypothesis is proposed:

**Hypothesis** **1.** *EOHSFV has a significant and positive effect on Chinese college students’ (a) OHK and (b) ROB.*

### 2.3. The Mediating Roles of OHK and ROB

Previous health literature indicated that exposure to prevention information may directly influence behaviors through psychological and cognitive factors [56,57,60]. Specifically, studies in line with this field have identified knowledge and optimistic bias as mediating mechanisms in the relationship between exposure to health information and individuals’ engagement in preventive behaviors (e.g., willingness to be vaccinated and influenza prevention). For instance, a study by Cameron et al. showed that exposure to influenza prevention information positively influenced individuals’ knowledge [56]. Additionally, Reinders et al. found that increased knowledge about influenza and vaccination was associated with higher annual vaccination rates [62]. Furthermore, Melki et al. reported that exposure to news regarding COVID-19 positively influenced individuals’ knowledge of preventive measures [57]. Chen et al. also found a positive association between knowledge of COVID-19 and individuals’ preventive behaviors [63].

ROB may mediate the relationship between EOHSFV and EOHP. Previous research has shown that individuals exposed to cancer risk and prevention messages perceive a stronger effect on themselves compared to others, a phenomenon known as ROB [60]. Furthermore, a prior study found that individuals with a greater sense of vulnerability (ROB) were less likely to intend to smoke cigarettes [64]. In addition, a previous study showed that exposure to information about radioactive pollution reduced the self–other perception gap, leading individuals to perceive the media’s influence on themselves as stronger than on others [65]. This psychological bias subsequently increased their intention to engage in radiation protection behaviors, such as taking iodide pills [65]. Based on these findings, the following hypotheses are proposed:

**Hypothesis** **2.** *OHK mediates the relationship between EOHSFV and Chinese college students’ EOHP.*

**Hypothesis** **3.** *ROB mediates the relationship between EOHSFV and Chinese college students’ EOHP.*

The existing literature primarily examined the direct and indirect effects of health information exposure on prevention behaviors in various contexts. However, a substantial body of research has also examined the influence of knowledge and optimistic bias on attitudes. For example, a previous study demonstrated that increased oral health knowledge improved individuals’ preventive attitudes, which subsequently promoted engagement in oral health behaviors [66,67]. Similarly, public health studies by Khan et al. found a significant and positive correlation between knowledge of Middle East Respiratory Syndrome (MERS) and attitudes, with further evidence suggesting that attitudes are critical determinants in strengthening preventive behaviors against MERS [68]. Additionally, a prior study found a negative association between perceptions of physical invulnerability and attitudes, which in turn increased college students’ willingness to engage in physical activity [69,70]. Similarly, another study confirmed that subjective overconfidence negatively affected individuals’ attitudes, which were further negatively associated with engagement in food safety behaviors [71,72]. These findings suggest that ROB and attitudes of Chinese college students may serially mediate the relationship between EOHPI and their engagement in oral health behaviors. Therefore, the following hypotheses are proposed:

**Hypothesis** **4.** *OHK and OHA play a serial mediating role in the relationship between EOHSFV and Chinese college students’ EOHP.*

**Hypothesis** **5.** *ROB and OHA play a serial mediating role in the relationship between EOHSFV and Chinese college students’ EOHP.*

**Figure 1:**
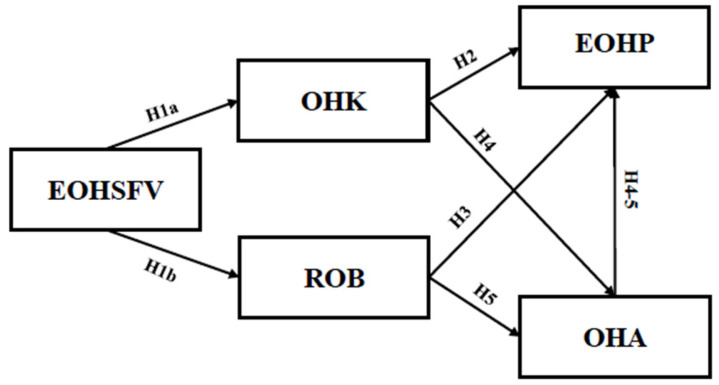
Model of predictors of the engagement in oral health prevention.

In summary, Figure 1 shows the extended theoretical model summarizing the hypotheses.

## 3. Methods

### 3.1. Sampling

The current study recruited college student users of short-form videos through WJX.cn, one of the most widely used online survey platforms in China, with nearly 260 million active users. This platform is commonly used to conduct health-related studies in China [73]. The sample for this study was randomly selected from the pool of the survey company. Informed consent was obtained on the first page of the online survey, and respondents were assured that their information would remain strictly confidential. The eligibility criteria for this study were as follows: (1) current enrollment as a college student, (2) age between 18 and 30 years, and (3) previous exposure to health-related short-form videos on Douyin. Participants were recruited from 18 January to 20 March 2024. Ethical approval for the present study was approved by the School of Journalism and Communication, Beijing Institute of Graphic Communication Academic Committee (SC20240118).

**Figure 2:**
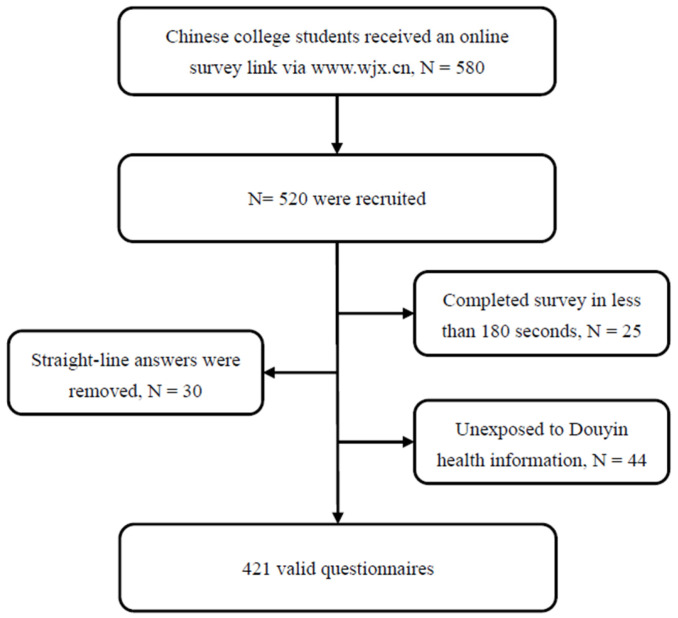
Respondent enrollment procedure.

Of the 580 potential respondents who received the survey link, a total of 520 participants completed the survey. To avoid biased sampling, non-probability sampling techniques were excluded. Following the methods of previous studies [74], a three-step data-cleaning process was implemented. First, 25 respondents were removed due to an insufficient survey completion time, defined as less than 180 s, also known as “speeders”. Second, 30 respondents were excluded for providing “straight-lined” answers. Finally, to ensure that respondents had been exposed to health-related information on Douyin, an initial filter question, “Have you ever been exposed to health-related information on Douyin?” was included. The enrollment procedure is shown in Figure 2.

Previous literature utilized G-Power 3.1 to evaluate the sample size, a method widely accepted in hypothesis-testing research [75]. Following the methodology established by previous scholars, with an effect size of 0.15, a power of 0.80, and four predictors, the minimum required sample size was determined to be 85. Additionally, to assess the sample size for mediation models, this study used Monte Carlo power analysis, a technique commonly used in health promotion and psychological research [76]. This analysis was conducted with 1000 replications and a significance level of 0.05. The results indicated that with a power of 0.80, the required minimum sample size was 104. Therefore, the sample size of the present study (N = 421) exceeded the calculated minimum sample size, confirming its adequacy for the intended analyses.

### 3.2. Measurements

The survey questionnaires were carefully developed based on previous measurements [37,77,78,79,80,81]. To enhance the accuracy and reliability, three focus groups and content and face validity methods were used, resulting in adjustments and elimination of items from the originally constructed five main measures. A pretest was then conducted with 15 volunteers to identify and correct any ambiguous or unclear items. The final measures, including their factor loadings, composite reliability (CR), and average variance extracted (AVE), are detailed in Appendix A.

In many cross-sectional survey studies, it is critical to assess and confirm the presence of common method bias (CMB) [82]. Avoiding CMB can improve convergent validity and reduce the likelihood of statistical error [83]. Therefore, in the present study, Harman’s single-factor test was applied to assess CMB. The results indicated that the single factor accounted for 35.45% of the variance, which is below the recommended threshold of 50%. Therefore, CMB did not appear to be a significant issue in this study.

Discriminant validity was assessed using the Fornell–Larcker criterion, which has been widely employed in previous studies [84,85]. The results indicated that each AVE value exceeded the corresponding correlations with other factors, thus confirming adequate discriminant validity for each factor (see Table 1).

### 3.3. Data Analysis Methods

For the current study, 421 valid samples were collected from 18 January to 20 March 2024. The data were analyzed using SPSS 25.0 with the following steps: First, reliability (Cronbach’s alpha) and validity (convergent and discriminant validity) were assessed to ensure the accuracy of the measurements [86]. Second, descriptive statistics (e.g., age, gender, and education) were calculated to profile the respondents. Finally, hierarchical regression analysis was conducted to test the hypothesized direct effects [72]. The PROCESS macro was performed to assess two mediated effects (H1 and H2) and two serial mediated relationships (H3 and H4). Additionally, bootstrapping was applied to obtain bias-corrected 95% confidence intervals, allowing for robust statistical inferences regarding specific mediator effects.

## 4. Results

### 4.1. Descriptive Data

A total of 421 valid respondent responses were collected. Table 2 shows the key demographic characteristics of the respondents. The majority were male (N = 213, 50.6%) and either undergraduates (N = 328, 77.9%) or postgraduates (N = 93, 22.1%). Additionally, the majority of respondents were between 22 and 25 years old (N = 167, 39.7%), followed by 18 to 21 years old (N = 136, 32.3%) and 26 to 30 years old (N = 118, 28.0%). Lastly, their monthly incomes showed that the majority earned between 4000 and 8999 RMB (N = 134, 31.9%) and between 9000 and 13,999 RMB (N = 116, 27.6%).

### 4.2. Hypothesis Testing

To test Hypothesis 1(a,b), hierarchical regression analysis was applied in SPSS 25.0 [87,88]. In the first block, demographic factors, including education, gender, and income, were entered as confounding variables. Secondly, EOHSFV was entered into the second block as an independent variable. Finally, OHK and ROB were entered as dependent variables. The results showed that the first regression model was statistically significant, explaining 19% of the variance (R^2^ = 0.19, *p* < 0.001), and the direct effect of EOHSFV on OHK was significant (β = 0.19, t = 7.47, *p* < 0.001). The second regression model was also significant, explaining 51% of the variance (R^2^ = 0.51, *p* < 0.001). The direct effect of EOHSFV on ROB showed a significant level (β = 0.46, t = 11.33, *p* < 0.001). Therefore, H1(a,b) were fully supported.

The Hayes’ PROCESS macro (model 4) was performed to assess the mediating hypotheses. We applied bootstrapping to obtain bias-corrected 95% confidence intervals to ensure statistical inferences regarding the mediated mechanisms [89]. In the first mediation model, OHK showed a positive effect on EOHP (β = 0.56, t = 8.96, *p* < 0.001). Furthermore, the indirect effect was significant (β = 0.10, t = 11.90, *p* < 0.001, 95% CI [0.08, 0.13]), confirming the mediating effect of OHK. In the second mediation model, ROB did not show a significant effect on EOHP (β = 0.04, t = 1.02, *p* > 0.05). The indirect effect was also insignificant (β = 0.10, t = 1.02, *p* > 0.05, 95% CI [−0.02, 0.10]), rejecting the mediating effect of ROB. In summary, Hypothesis 2was supported, while Hypothesis 3 was not.

**Figure 3:**
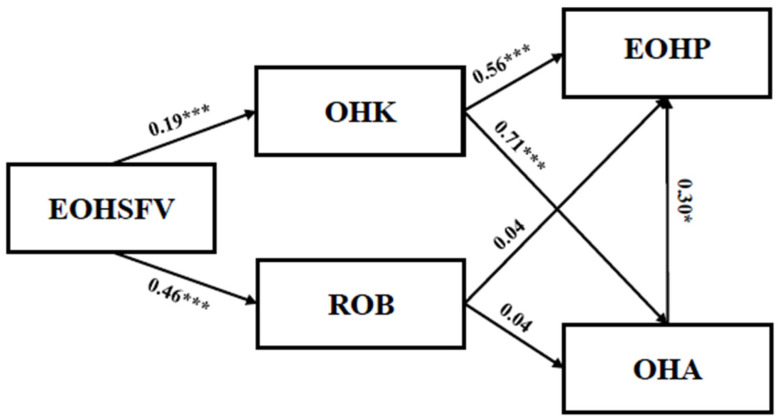
The impact of factors predicting engagement in oral health prevention. * *p* < 0.05, and *** *p* < 0.001.

The serial mediation hypotheses (Hypotheses 4 and 5) were tested using the Hayes PROCESS macro (model 6). Results for the first serial mediation model indicated that EOHSFV had a positive effect on OHK (β = 0.18, t = 7.11, *p* < 0.001). OHK had a positive effect on OHA (β = 0.71, t = 18.18, *p* < 0.001) and EOHP (β = 0.35, t = 4.22, *p* < 0.001). In addition, OHA positively predicted EOHP (β = 0.30, t = 3.90, *p* < 0.01), and the serial mediation effect was significant (β = 0.04, t = 11.42, *p* < 0.01, 95% CI [0.01, 0.10]). Thus, Hypothesis 4 was supported. The results of the second serial mediation model indicated that EOHSFV had a positive effect on ROB (β = 0.44, t = 11.33, *p* < 0.001). However, ROB showed an insignificant effect on OHA (β = 0.04, t = 1.15, *p* > 0.05) and EOHP (β = 0.02, t = 0.61, *p* > 0.05). In addition, OHA positively predicted EOHP (β = 0.51, t = 8.73, *p* < 0.001), but the serial mediation effect was not significant (β = 0.01, t = 10.41, *p* > 0.05, 95% CI [−0.01, 0.03]). In conclusion, Hypothesis 4 was supported, while Hypothesis 5 was not. Table 3 provides a summary of the hypotheses and hypothesis testing results. Figure 3 presents the standardized coefficients and significance for each path in the hypothesized model.

## 5. Discussion

The main objective of this research was to examine the effects of exposure to oral health short-form videos via Douyin on engagement in oral health prevention in the Chinese context. Additionally, the study aimed to examine the mediating mechanisms through which psychological and cognitive factors influence preventive behaviors among young adults in China. While previous studies have examined various health behaviors using the KAP theoretical model, this study extended the model by including the role of optimistic bias in promoting engagement in oral health prevention among Chinese young adults. The findings enhanced the explanatory power and applicability of the theory in the context of oral health prevention in China. Furthermore, the results provide valuable insights for the National Health Commission (NHC) and other health authorities to better understand the intricate mechanisms involved, thereby improving public health strategies and designing more persuasive oral health short-form video promotions.

In the context of China, Douyin, one of the most popular media platforms, has played an important role in improving people’s oral health knowledge. The present study found that exposure to oral health short-form videos significantly contributed to the oral health knowledge of Chinese college students. This finding is consistent with previous studies indicating that increased exposure to information about influenza is correlated with improved knowledge of influenza vaccination [56]. In addition, the short-form videos on Douyin were also recognized as a key influence in increasing reversed optimistic bias regarding oral health among Chinese college students. This finding is consistent with previous research suggesting that individuals exposed to health messages tend to perceive these messages as having a greater impact on themselves than on their peers. Specifically, individuals are more likely to assume that they are at greater risk of experiencing adverse health outcomes, such as cancer, than others [60].

The results showed that oral health knowledge mediated the relationship between exposure to oral health short-form videos and engagement in oral health prevention among Chinese college students, supporting Hypothesis 2. This is consistent with previous studies, showing that exposure to health-related information, including influenza prevention, increased individuals’ prevention-related knowledge [56]. Consequently, this increase in knowledge had a positive impact on their vaccination uptake [62]. Similarly, knowledge has been identified as an important mediator in the relationship between exposure to health information and the effectiveness of individuals’ influenza prevention measures [57,63].

An unanticipated finding was that reversed optimistic bias did not mediate the relationship between exposure to oral health short-form videos and engagement in oral health prevention among Chinese college students (Hypothesis 3). This is in contrast to the findings of previous studies [60,64,65]. For example, one study found that frequent exposure to cancer prevention information increased individuals’ sense of vulnerability (also known as reversed optimistic bias) and further increased their intention to avoid smoking cigarettes [60]. This can be explained by the fact that health content on Douyin tends to emphasize an entertaining atmosphere rather than educational content or communication of significant health threats. Such a media effect may reduce its ability to influence psychological biases, such as the optimistic bias. As a result, college students may believe that they are less likely to experience negative outcomes, weakening the potential impact of health messages. In addition, due to the push algorithm settings on Douyin, individuals are exposed to a wide range of information, which may weaken the impact of oral-health-related content. Simultaneous exposure to both trending short-form videos and health-related content makes it difficult for users to focus exclusively on oral health knowledge and prevention messages, further reducing the impact on susceptibility to risk or negative outcomes among Chinese college students.

Contrary to findings in other studies, reversed optimistic bias and oral health attitudes did not demonstrate a serial mediation mechanism in the relationship between exposure to oral health short-form videos and engagement in oral health prevention among Chinese college students. This finding contradicts previous literature suggesting that physical invulnerability positively influences health attitudes. Furthermore, these improved attitudes have been shown to increase engagement in physical activity [70]. One possible reason for this difference is that attitudes may not only directly enhance health prevention behaviors, but may also influence health belief factors, such as perceived health benefits. When individuals are exposed to health messages, their perceived benefits increase significantly, helping them to overcome uncertainty and better understand the risks associated with health prevention [90]. For example, a previous study examined the determinants that influence individuals’ engagement in healthy meal consumption and revealed that attitude was a key mediator in the relationship between perceived health benefits and healthy meal consumption [91]. Considering this prior empirical evidence, future studies are encouraged to further explore whether a triple serial mediation mechanism exists (exposure to oral health short-form videos → reversed optimistic bias → oral health attitude → perceived health benefits → engagement in oral health prevention).

The theoretical contributions of the current study are two-fold. First, this study went beyond previous literature on individual engagement in health prevention by specifically examining the effect of short-form video exposure on engagement in oral health prevention among college students in the context of China. While previous research based on the KAP model has examined health prevention from a perspective that includes key antecedents, such as social media use [35,92,93], these studies did not address how health content on cutting-edge media platforms can significantly influence knowledge, attitudes, and engagement in prevention behaviors. In doing so, this study extended the theoretical understanding of how emerging media technologies contribute to health behavior changes. Second, this study extended the KAP model by incorporating optimistic bias theory to examine the impact of short-form videos on Chinese college students’ engagement in oral health prevention. This pioneering study improved the KAP model by increasing its explanatory power and providing a more comprehensive framework that addressed the limitations identified in previous research. As expected, the study found that exposure to oral health short-form videos not only increased oral health knowledge, but also influenced reversed optimistic bias. Based on these empirical findings, future research is encouraged to apply the extended KAP model in various health prevention contexts.

It is vital to suggest practical implications for the design of campaigns in oral health contexts. The current study found that reversed optimistic bias did not mediate the relationship between exposure to oral health short-form videos and engagement in oral health prevention among Chinese college students. This suggests that simply creating content that increases individuals’ sense of vulnerability is not sufficient. For example, a study showed that using gamification strategies in nutrition media had a significant impact on individuals’ healthy food intake [94]. Therefore, health content creators are encouraged to develop more diverse and interactive types of health content. While exposure to oral health short-form videos accounted for 19% of the variance in oral health knowledge (R^2^ = 0.19, *p* < 0.001), these results should be treated with caution. A significant factor contributing to this modest impact is the growing presence of unverified health experts in China, who create and disseminate oral health content without proper validation. Research indicates that such unverified content often attracts more views and shares than material produced by medical professionals [95]. This exposure to inaccurate health information can lead to misguided health practices [96]. Therefore, we recommend that short-form video platforms collaborate with health organizations and experts to produce high-quality, credible oral health content.

The following limitations should be considered in future research. First, the current study did not consider other types of cognitive biases, such as the “illusion of knowledge”, a bias in which individuals perceive themselves as well informed when they are not [97]. Previous research has examined how exposure to media messages influences people’s heuristic choices or accurate evaluations of knowledge in different contexts [98]. Considering that oral health content on Douyin may also contribute to this bias, future studies may benefit from investigating the illusion of knowledge in the context of oral health promotion on social media. Second, the measure of reversed optimistic bias was derived from an existing scale [78]. Content and face validity tests were conducted to systematically validate the measurement for accuracy and reliability. Although the final measure met the required standards, the limited number of items presented a challenge in fully capturing the complexity of reversed optimistic bias. In addition, more precise measurement methods, such as focus groups, the Delphi method, and pilot testing, have not yet been fully applied. Therefore, future studies are encouraged to use these methods to improve the measurement process. In addition, as digital health platforms are increasingly recognized as essential resources for health promotion, a key implication is the necessity of delivering more effective health content to enhance individuals’ ability to comprehend and apply health-related information [99]. To achieve this, the content must be carefully tailored and presented in an appropriate format [100]. Furthermore, previous studies have shown that the impact of media platforms on health communication may vary from country to country [101]. That is to say, limited findings from certain studies may not be generalizable to all types of digital health platforms or applicable across different countries. Considering the persistent gap in exploring global digital health platforms and their impact, future research is strongly encouraged to investigate the complex relationships between exposure to digital health content and subsequent changes in health behavior.

## 6. Conclusions

The present study examined the relationship between exposure to oral health short-form videos on Douyin and engagement in oral health prevention among Chinese college students (aged 18 to 24 years), a demographic known for higher oral health risks due to irregular and unhealthy habits. The results revealed the significant explanatory power of the extended KAP theory model in engagement in oral health prevention research. Based on these findings, the analysis of direct, mediated, and serially mediated pathways has enhanced our understanding of these complex influences, contributing to the improvement of public health strategies and the design of more effective oral health short-form video promotions.

## Figures and Tables

**Table 1:** Fornell–Larcker criterion.

Variables	1	2	3	4	5
EOHSFV	0.88				
ROB	0.48 **	0.86			
OHK	0.32 **	0.24 **	0.71		
OHA	0.31 **	0.20 **	0.70 **	0.81	
EOHP	0.57 **	0.31 **	0.49 **	0.48 **	0.85

** *p* < 0.01.

**Table 2:** Key demographic characteristics of the survey participants.

Variables	Item	Count	Percentage
Sex	Female	208	49.4%
	Male	213	50.6%
Education level	Undergraduate	328	77.9%
	Postgraduate	93	22.1%
Age	18–21 years old	136	32.3%
	22–25 years old	167	39.7%
	26–30 years old	118	28.0%
Monthly household income (RMB)	1000–3999	68	16.2%
	4000–8999	134	31.9%
	9000–13,999	116	27.6%
	>14,000 RMB	103	24.5%
	Total	421	100%

**Table 3:** Summary of hypothesis testing results.

Hypotheses	Relationship	Result
Hypothesis 1	EOHSFV has a significant and positive effect on Chinese college students’ (a) OHK and (b) ROB.	Supported
Hypothesis 2	OHK mediates the relationship between EOHSFV and Chinese college students’ EOHP.	Supported
Hypothesis 3	ROB mediates the relationship between EOHSFV and Chinese college students’ EOHP.	Rejected
Hypothesis 4	OHK and OHA play a serial mediating role in the relationship between EOHSFV and Chinese college students’ EOHP.	Supported
Hypothesis 5	ROB and OHA play a serial mediating role in the relationship between EOHSFV and Chinese college students’ EOHP.	Rejected

## Data Availability

The original data are provided by the authors. If there are relevant research needs, the data can be obtained by sending an email to the corresponding author. Please indicate the purpose of the research and the statement of data confidentiality in the email.

## Appendix A

**Table A1:** Items and Measurement Model Assessment.

Construct	Source	Items	Loading	α	CR	AVE
Exposure to oral health short-form videos (EOHSFV)	Liu et al. (2020) [77]	Dental health practical tips short videos.	0.91	0.87	0.99	0.77
		Dental drama short videos.	0.90			
		Oral health popular science short videos (e.g., causes of cavities and wisdom teeth).	0.90			
		Dental imaging short videos (e.g., X-rays).	0.87			
		Oral care product introduction and marketing short videos.	0.82			
Reversed optimistic bias (ROB)	van der Meer et al. (2023) [78]	I may be more susceptible to oral diseases than others.	0.82	0.90	0.97	0.75
		Oral diseases may disrupt my life and work rhythm compared to others.	0.91			
		Oral diseases may lead to more severe health consequences for me compared to others.	0.90			
		Oral diseases may have more severe economic consequences for me than others.	0.84			
Oral health knowledge (OHK)	Tadin et al. (2022) [79]	In order to prevent tooth decay, it is necessary to brush teeth frequently (with emphasis on the crown).	0.66	0.87	0.98	0.50
		You should brush your teeth every morning and before bed every night.	0.65			
		Plaque can lead to gum disease and cavities.	0.77			
		Gum bleeding is a sign of gum disease.	0.76			
		Diabetes increases the risk of gum disease.	0.68			
		It is especially important to gargle at least once a day.	0.74			
		Smoking/vaping is associated with the occurrence of periodontitis.	0.67			
		It is necessary to visit the dentist regularly to check the condition of the mouth and to have a professional cleaning.	0.75			
		Maintain oral health by eating a strictly balanced diet and limiting sweets (including sugary drinks).	0.70			
Oral health attitude (OHA)	Francis et al. (2004) [80]; Ajzen (2006) [102]; Ho et al. (2015) [103]	I believe that oral health practices are pleasant.	0.81	0.81	0.96	0.66
		I believe that oral health practices are beneficial.	0.87			
		I believe that oral health practices are good.	0.81			
		I believe that oral health practices are important.	0.73			
Engagement in oral health prevention (EOHP)	Brennan et al. (2010) [81]; Zheng et al. (2021) [37]	I avoid sweets, fizzy drinks, tobacco, and alcohol to prevent oral diseases.	0.78	0.81	0.94	0.73
		I have regular oral care (e.g., brushing my teeth, removing tartar, and removing plaque).	0.90			
		I visit the dentist regularly to prevent oral diseases.	0.88			
